# Quercetin Mitigates Oxidative Stress-Induced Premature Senescence in SH-SY5Y Neuronal-like Cells

**DOI:** 10.3390/ijms27135759

**Published:** 2026-06-26

**Authors:** Federica Lina Salamone, Maria Sofia Molonia, Santi Trischitta, Antonella Saija, Francesco Cimino, Antonio Speciale

**Affiliations:** 1 Department of Chemical, Biological, Pharmaceutical and Environmental Sciences, University of Messina, Viale F. Stagno D’Alcontres 31, 98166 Messina, Italy; federica.salamone@studenti.unime.it (F.L.S.); mariasofia.molonia@unime.it (M.S.M.); santi.trischitta@studenti.unime.it (S.T.); antonina.saija@unime.it (A.S.); francesco.cimino@unime.it (F.C.); 2 “Prof. Antonio Imbesi” Foundation, University of Messina, 98100 Messina, Italy

**Keywords:** quercetin, oxidative stress, neuronal senescence, SH-SY5Y cells, Nrf2 signaling, senescence-modulating activity, SASP

## Abstract

Cellular senescence is a biological process involved in aging and neurodegenerative disease progression, characterized by cell-cycle arrest, oxidative stress, persistent DNA damage, and development of a pro-inflammatory senescence-associated secretory phenotype (SASP). While replicative senescence results from exhaustion of cellular proliferative capacity, stress-induced premature senescence (SIPS) can be induced by multiple triggers, contributing to various pathological conditions. Among compounds reported to modulate cellular senescence, quercetin (QUE), a dietary flavonoid with antioxidant and anti-inflammatory properties, has emerged as a promising modulator of senescence-related pathways. This study investigated the protective effects of QUE against oxidative stress-induced senescence in SH-SY5Y neuronal-like cells. Cells were exposed to H_2_O_2_ (25 μM) to trigger early SIPS and subsequently treated with QUE (2.5 and 5 μM) for 24 h. H_2_O_2_ induced a senescence-like phenotype characterized by increased senescence-associated β-galactosidase (SA-β-gal) activity, lamin-B1 depletion, activation of p53/p21 pathway, modulation of Bcl-2/Bax ratio, and upregulation of SASP mediators, including NF-κB, *MCP-1* and *PAI-1*. QUE treatment significantly attenuated these markers in a dose-dependent manner. These effects were associated with the enhancement of Nrf2-linked antioxidant responses, indicating a potential contribution to QUE senescence-modulating properties. These findings support the potential of QUE as a senescence-modulating compound against oxidative stress-induced neuronal senescence through the modulation of key molecular pathways, along with the enhancement of Nrf2-associated antioxidant defenses.

## 1. Introduction

Cellular senescence, first identified by Hayflick and Moorhead [1], was originally regarded primarily as a characteristic feature of aging, since normal human diploid cell strains undergo a finite number of population doublings before entering a state of irreversible growth arrest, a phenomenon now known as “Hayflick’s limit” [2]. Today, cellular senescence is recognized as a complex and dynamic biological process, characterized by a stable cell cycle arrest that serves as a protective mechanism in response to various stressors, including telomere attrition, mitochondrial dysfunction, DNA damage, oxidative stress, oncogenic activation, and inactivation of tumour suppressor genes [3,4,5]. In addition to their irreversible proliferative arrest, senescent cells undergo multiple phenotypic and functional alterations, such as metabolic reprogramming, chromatin remodeling, sustained activation of DNA damage response (DDR), and modulation of apoptosis and autophagy pathways [6,7]. In particular, a typical feature of senescent cells is the enhancement of lysosomal activity [8], and the induction of the senescence-associated secretory phenotype (SASP), a complex and heterogeneous profile of pro-inflammatory cytokines, chemokines, growth factors, and proteases that profoundly influence the surrounding tissue microenvironment [9].

In addition to replicative senescence driven by telomere shortening, a senescence-like state can also be rapidly induced by sub-lethal stressors, a process referred to as stress-induced premature senescence (SIPS). SIPS exhibits key molecular and functional features of cellular senescence and can arise in response to diverse insults, including oncogenic activation, mitochondrial dysfunction, viral infection, and genotoxic or oxidative stress [10,11].

Senescence is commonly associated with age-related diseases, such as atherosclerosis, diabetes, and lung disease [12,13], yet senescent cells also influence several physiological and pathological processes, including embryogenesis, tissue remodeling, wound healing, tumour suppression, and tumorigenesis [14,15,16].

Cellular senescence is increasingly recognized as a key process involved in the onset and progression of neurodegenerative diseases (NDs) [17]. Senescent neuronal cells are closely associated with the abnormal production and accumulation of toxic protein aggregates, such as β-amyloid, Tau, and α-synuclein, which in turn promote neuronal dysfunction and death, leading to disorders such as Alzheimer’s and Parkinson’s diseases [18,19]. Furthermore, senescent cells can disrupt the homeostasis of the neuronal microenvironment through paracrine signaling pathways, thereby enhancing senescence-related effects and damaging nearby healthy cells [20]. Also, the shift to a senescent state in microglia, astrocytes, and cerebral vascular endothelial cells can contribute to neuroinflammation, altering the structure of the blood-brain barrier and impairing neuroregenerative mechanisms [21]. Simultaneously, the development of NDs can increase the generation of senescent cells, further amplifying neuroinflammatory and neurotoxic processes. For this reason, targeting cellular senescence represents a promising therapeutic strategy for NDs, with the aim of preventing and mitigating senescence-driven neurodegeneration.

The “senotherapeutic” strategies are generally divided into two main categories: senolytics, which selectively eliminate senescent cells, and senomorphics, which modulate the SASP profile [22]. Approaches such as dietary interventions and pharmacological agents, including metformin, the mTOR inhibitor rapamycin, and the sirtuin agonist resveratrol, have shown protective activities against cellular senescence [23]. More recently, SASP inhibitors and senolytic compounds have emerged as promising agents to improve age-related pathologies [24]. Among these, quercetin (QUE), a naturally occurring flavonoid with well-known antioxidant and anti-inflammatory properties [25,26], has been identified as a potential senolytic agent, promoting the elimination of senescent cells by inducing apoptosis and reducing SASP-mediated inflammation [27,28,29,30]. Moreover, a recent pilot clinical study evaluating the senolytic combination dasatinib plus QUE in older adults at risk for Alzheimer’s disease provides early clinical evidence supporting the translational relevance of QUE-based senescence-modulating strategies [31]. To the best of our knowledge, while individual aspects of oxidative stress-induced senescence have been investigated, a combined evaluation of senescence markers, antioxidant response, and inflammatory signaling in neuronal-like models remains limited, particularly in the context of early senescence-like responses.

Based on this evidence, we established an in vitro model of stress-induced premature senescence (SIPS) [11] in SH-SY5Y neuronal-like cells by exposing them to H_2_O_2_-induced oxidative stress, resulting in an early senescence-like phenotype, to evaluate the impact of QUE on senescence-associated alterations in these cells. To investigate how QUE modulates key features of H_2_O_2_-induced senescence, we analysed key senescence markers, including cell cycle regulators, apoptotic pathways, SASP-related factors, and antioxidant stress-response pathways.

## 2. Results

### 2.1. Quercetin Attenuates Senescence-Associated β-Galactosidase Activity

The first and most widely used biomarker to identify senescent cells in both in vitro and in vivo models is the accumulation of the lysosomal enzyme known as senescence-associated-β-galactosidase (SA-β-gal) [22]. β-galactosidase is a lysosomal hydrolase that, when highly concentrated, can promote the disintegration of proteoglycans and the structural alteration of basement membranes through the hydrolysis of glycosidic bonds [32]. This enzymatic activity is markedly amplified in senescent cells due to an increase in lysosomal content [6], and is generally absent in pre-senescent, quiescent, immortal or transformed cells. β-gal activity is typically detected at a suboptimal pH of 6.0, due to its marked overproduction in senescent cells [33].

**Figure 1:**
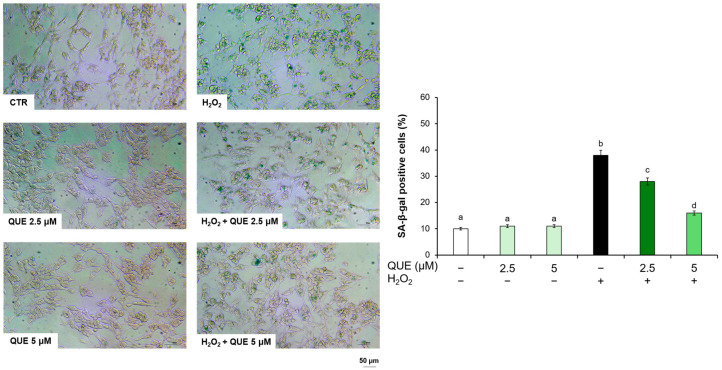
Effects of QUE on senescence-associated β-galactosidase activity. SH-SY5Y cells were treated with H_2_O_2_ (25 μM) for 1 h, followed by incubation with QUE (2.5 and 5 μM) for 24 h. Cells treated with the vehicle alone (DMSO 0.01%) were used as controls (CTR). Representative images of SA-β-gal-stained cells show blue cytoplasmic deposits indicative of senescence. Images were acquired at 20× magnification using an optical microscope. The bar graph reports the percentage of SA-β-gal-positive cells (mean ± SD; n = 3 independent experiments). Means sharing the same letter are not significantly different from each other (*p* > 0.05).

As shown in Figure 1, SH-SY5Y cells exposed to H_2_O_2_ displayed a significant increase in SA-β-gal-positive cells compared to the untreated CTR cells. Conversely, treatment with QUE at both concentrations significantly reduced the percentage of SA-β-gal-positive cells in a dose-dependent manner, indicating its ability to counteract senescence-associated lysosomal alterations.

### 2.2. Quercetin Attenuates Senescence-Associated Lamin-B1 Depletion

Senescent cells exhibit important morphological changes, including enlarged and irregular nuclei and chromatin reorganization. Specifically, lamin-B1 is the main scaffolding protein of the nuclear lamina, which regulates nuclear architecture, chromatin organization, and transcriptional activity [34,35]. Loss of lamin-B1 in senescent cells compromises nuclear integrity, leading to the accumulation of cytoplasmic DNA and promoting the formation of senescence-associated heterochromatin foci (SAHF) as well as the activation of senescence-associated genes [36,37,38].

**Figure 2:**
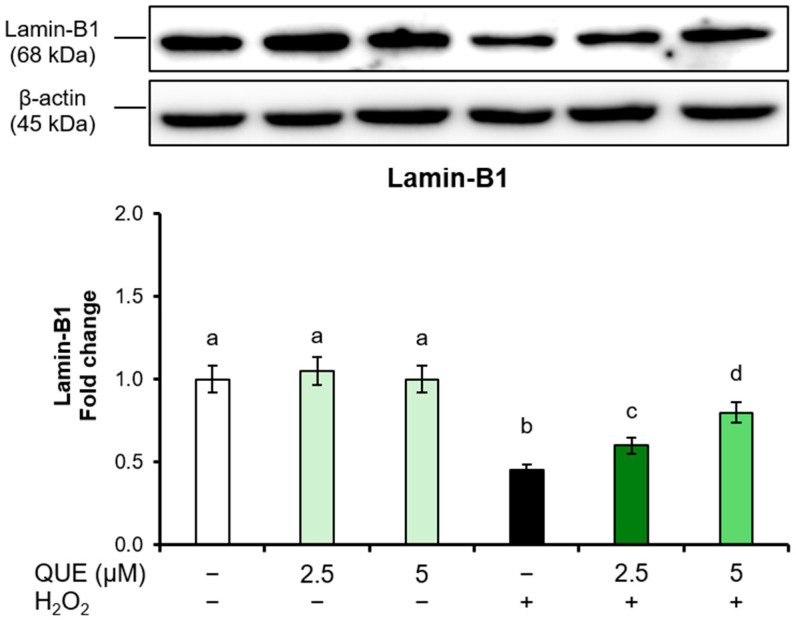
QUE modulation of lamin-B1 levels. SH-SY5Y cells were treated with H_2_O_2_ (25 μM) for 1 h, followed by incubation with QUE (2.5 and 5 μM) for 24 h. Cells treated with the vehicle alone (DMSO 0.01%) served as controls. Lamin-B1 protein levels were determined in whole cell lysates and normalized to β-actin levels. Results are reported as fold change relative to control and are expressed as mean ± SD of three independent experiments, each performed in triplicate. Means sharing the same letter are not significantly different from each other (*p* > 0.05).

To investigate whether QUE modulates senescence-associated nuclear changes, we evaluated lamin-B1 protein levels. SH-SY5Y cells exposed to H_2_O_2_ showed a significant reduction in lamin-B1 levels compared to CTR cells. Treatment with QUE restored lamin-B1 levels in a dose-dependent manner, indicating its capability to attenuate senescence-related lamin-B1 depletion and nuclear lamina alterations. QUE alone did not induce any significant alteration in basal lamin-B1 levels (Figure 2).

### 2.3. Quercetin Alleviates Senescence-Associated Cell Cycle Arrest

The cell cycle is based on carefully coordinated processes that result in cell division, essential for the development, growth and survival of eukaryotic organisms [39]. Exposure to various factors, such as DNA damage caused by telomere attrition, oncogenic signals, or oxidative stress, triggers the initiation of the p53-mediated signaling cascade.

As a transcription factor, activated p53 [phospho-p53 (Ser15)] subsequently upregulates p21^Waf1/Cip1^, which inhibits cyclin-dependent kinase 1, 2, 4, and 6 (CDKs), preventing the progression from the G1 to the S phase of the cell cycle [40,41]. This event prevents the propagation of damaged DNA and significantly contributes to the onset and maintenance of the senescent phenotype [42].

To investigate the senescence-modulating activity of QUE against H_2_O_2_-induced senescence-linked response in our SH-SY5Y model, we analyzed the modulation of key cell cycle regulators. Exposure to H_2_O_2_ markedly increased phospho-p53 (Ser15) and p21^Waf1/Cip1^ protein levels compared to untreated CTR cells, confirming the induction of a senescence-like phenotype. Consistently, SRB assay analysis showed a slight, non-significant reduction in cell proliferation following H_2_O_2_ exposure, suggesting a mild cytostatic trend under these conditions. QUE treatment did not induce a decrease in cell number, although a tendency toward recovery was observed at both concentrations tested, with values approaching control levels at 5 μM (Figure S3).

**Figure 3:**
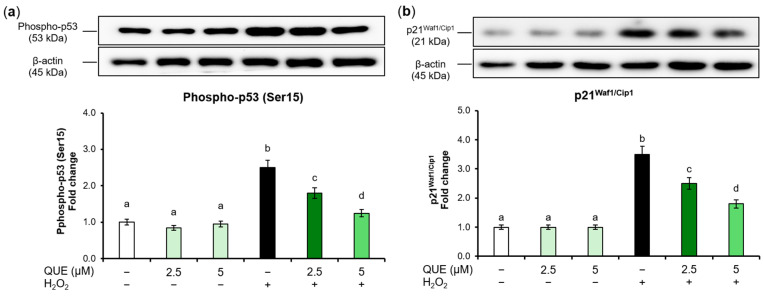
QUE effects on cell cycle mediators. SH-SY5Y cells were treated with H_2_O_2_ (25 μM) for 1 h, followed by incubation with QUE (2.5 and 5 μM) for 24 h. Cells treated with the vehicle alone (DMSO 0.01%) served as controls. (**a**) phospho-p53 (Ser15) and (**b**) p21^Waf1/Cip1^ protein levels were determined in whole cell lysates and normalized to β-actin levels. Results are reported as fold change relative to control and are expressed as the mean ± SD of three independent experiments, each performed in triplicate. Means sharing the same letter are not significantly different from each other (*p* > 0.05).

**Figure 4:**
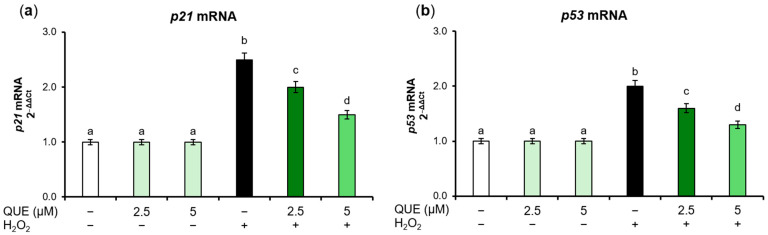
QUE effects on cell cycle mediators at the transcriptional level. SH-SY5Y cells were treated with H_2_O_2_ (25 μM) for 1 h, followed by incubation with QUE (2.5 and 5 μM) for 24 h. Cells treated with the vehicle alone (DMSO 0.01%) served as controls. *p21* (**a**) and *p53* (**b**) gene expression levels are expressed as 2^−ΔΔCt^ and normalized to control cells. GAPDH was used as a housekeeping gene. Results are reported as fold change relative to control and are expressed as the mean ± SD of three independent experiments, each performed in triplicate. Means sharing the same letter are not significantly different from each other (*p* > 0.05).

In contrast, under these conditions, treatment with QUE (2.5 and 5 μM) resulted in a dose-dependent reduction in both proteins, indicating that QUE attenuates the senescence-associated cell cycle arrest and partially restores cell cycle progression (Figure 3a,b). A similar trend was also observed at the transcriptional level, as shown by the expression values of *p53* and *p21* genes reported in Figure 4a,b. Treatment with QUE alone did not cause any significant change in the basal levels of the markers analyzed.

### 2.4. Quercetin Modulates Apoptosis-Related Signaling in Senescence-like Responses

Cellular senescence and apoptosis represent two distinct yet highly interconnected physiological responses to various stressors, including DNA damage, oxidative stress, and oncogene activation, both aimed at preserving cellular homeostasis [5]. Apoptosis leads to the programmed death of irreversibly damaged cells through activation of the caspase cascade, whereas senescence induces a stable cell cycle arrest, resulting in the accumulation of non-dividing but metabolically active cells [43,44]. Senescent cells upregulate anti-apoptotic mediators, particularly members of the Bcl-2 family (Bcl-2, Bcl-W, and Bcl-XL), which confer resistance to apoptosis and contribute to chronic inflammation and tissue dysfunction [45,46]. In addition, p53 and p21, which are involved in cell cycle regulation, may also inhibit apoptosis, thereby promoting senescence [5]. Consistent with this evidence, numerous senescence-modulating agents have been described to modulate pathways associated with cell survival and death, including apoptosis-related signaling [22].

**Figure 5:**
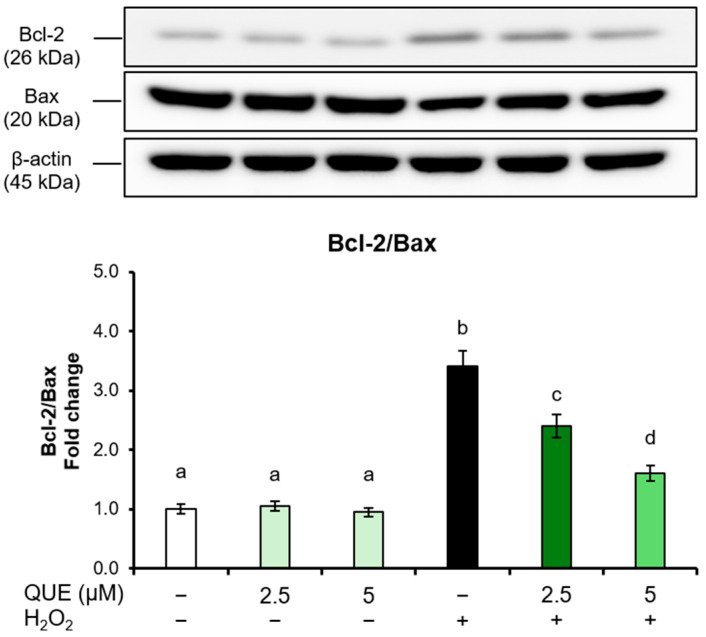
QUE effect on cellular apoptosis. SH-SY5Y cells were treated with H_2_O_2_ (25 μM) for 1 h, followed by incubation with QUE (2.5 and 5 μM) for 24 h. Cells treated with the vehicle alone (DMSO 0.01%) were used as controls. Bcl-2 and Bax protein levels were determined in whole lysates and normalized to β-actin levels. Bcl-2/Bax ratios are reported as fold change relative to controls and are expressed as mean ± SD of three independent experiments each performed in triplicate. Means sharing the same letter are not significantly different from each other (*p* > 0.05).

To investigate the effect of QUE on senescence-associated apoptosis-related signaling, we analysed the levels of two key members of the Bcl-2 family. The pro-apoptotic protein Bax promotes cytochrome *c* release from mitochondria, leading to apoptosome formation and subsequent activation of executioner caspases. In contrast, the anti-apoptotic protein Bcl-2 prevents this process by stabilizing mitochondrial membrane integrity and thereby promoting cell survival [47,48]. Exposure to H_2_O_2_ was associated with a change in apoptosis-related signaling, as evidenced by an increased Bcl-2/Bax ratio. Conversely, treatment with QUE decreased the Bcl-2/Bax ratio in a dose-dependent manner, indicating a shift in apoptosis-related signaling (Figure 5). Importantly, SRB assay data indicated only a slight reduction in cell proliferation following H_2_O_2_ exposure, consistent with a non-cytotoxic condition, with a tendency toward mild cytostatic effects. Under these conditions, QUE treatment did not induce a decrease in cell number (Figure S3), supporting the interpretation that the observed modulation of the Bcl-2/Bax ratio reflects changes in apoptosis-related signaling rather than apoptosis induction or non-specific cytotoxicity.

### 2.5. Quercetin Ameliorates SASP Condition

**Figure 6:**
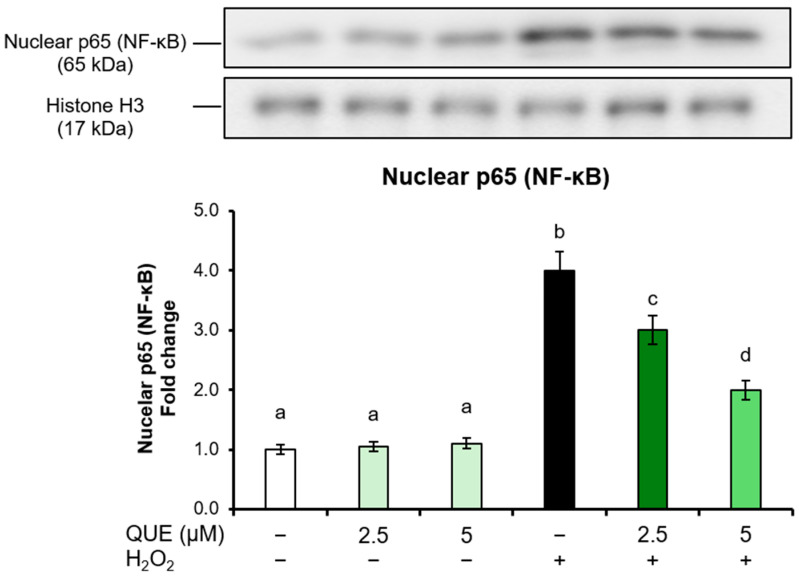
QUE anti-inflammatory activity. SH-SY5Y cells were treated with H_2_O_2_ (25 μM) for 1 h, followed by incubation with QUE (2.5 and 5 μM) for 24 h. Cells treated with the vehicle alone (DMSO 0.01%) were used as controls. Nuclear p65 (NF-κB) protein levels were determined in nuclear extracts and normalized to histone H3 levels. Results are reported as fold change relative to controls and are expressed as mean ± SD of three independent experiments, each performed in triplicate. Means sharing the same letter are not significantly different from each other (*p* > 0.05).

The most notable consequence of cellular senescence is the production and secretion of various mediators, including cytokines, chemokines, proteases, growth factors, and other signaling molecules, which form the SASP [6,49]. The SASP promotes the progression and maintenance of senescence, the development of chronic inflammation, and altered immune regulation in senescent tissues, with pathological manifestations [50]. Furthermore, the SASP is regulated by multiple signaling pathways, such as the nuclear factor kappa-B (NF-κB), p38 mitogen-activated protein kinase (MAPK), and mechanistic target of rapamycin (mTOR) [51]. In particular, the NF-κB signaling pathway plays a pivotal role in regulating the transcription of pro-inflammatory mediators. Upon activation, the p65/p50 NF-κB complex is released from its cytoplasmic inhibitor IκBα and translocates into the nucleus, where the p65 subunit binds to specific DNA sequences, initiating the transcription of several genes involved in cell survival, inflammation, senescence, and immune response [52]. In our experimental model, H_2_O_2_ exposure triggered a significant nuclear translocation of the p65 subunit (NF-κB) in SH-SY5Y cells, indicating the activation of a SASP-associated inflammatory response. Conversely, QUE treatment reduced nuclear p65 levels in a dose-dependent manner, demonstrating its ability to attenuate senescence-related NF-κB activation (Figure 6).

Furthermore, various NF-κB-dependent factors, such as monocyte chemoattractant protein-1 (MCP-1) and plasminogen activator inhibitor-1 (PAI-1), are closely related to the SASP. MCP-1 is a secreted chemokine that contributes to the recruitment of monocytes and lymphocytes to senescent tissues [53] and enhances senescence-associated signaling by increasing the protein levels of p53 and p21 via ROS or p38-MAPK signaling [54]. PAI-1 modulates extracellular matrix remodeling, strengthens cell cycle arrest by stabilizing p21 and modulating growth factor pathways, and promotes senescence propagation via paracrine signaling in tissues [55]. Since PAI-1 is transcriptionally induced by p53 [56] and acts as a key mediator of p21-dependent cell-cycle arrest and senescence maintenance [57], its upregulation reflects not only the inflammatory branch of the SASP but also the engagement of core senescence programs.

**Figure 7:**
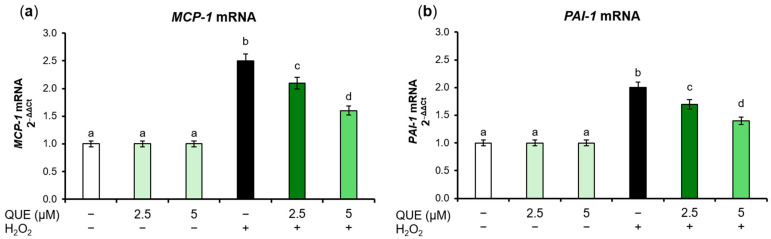
QUE anti-inflammatory activity at the transcriptional level. SH-SY5Y cells were treated with H_2_O_2_ (25 μM) for 1 h, followed by incubation with QUE (2.5 and 5 μM) for 24 h. Cells treated with the vehicle alone (DMSO 0.01%) were used as controls. *MCP-1* (**a**) and *PAI-1* (**b**) gene expression levels are expressed as 2^−ΔΔCt^ and normalized to control; GAPDH was used as the housekeeping gene. Results are reported as fold change relative to CTR and are expressed as mean ± SD of three independent experiments, each performed in triplicate. Means sharing the same letter are not significantly different from each other (*p* > 0.05).

To evaluate the inflammatory component of the senescent phenotype, we measured the expression of *MCP-1* and *PAI-1* genes. As shown in Figure 7, H_2_O_2_ exposure induced a clear upregulation of both markers compared to control cells. QUE treatment produced a modest reduction in the expression of the SASP-associated genes compared with H_2_O_2_-treated cells. Although the decrease was limited, both markers showed a downward trend, suggesting a partial attenuation of the inflammatory component of the senescence-like phenotype.

### 2.6. Quercetin Activates the Nrf2/ARE Pathway in SH-SY5Y Cells

Oxidative stress, mitochondrial dysfunction, chronic inflammation, and the dysregulation of cellular autophagy and apoptosis are key pathophysiological processes contributing to the onset and progression of age-related diseases [58,59]. In this context, the nuclear factor-erythroid 2-related factor 2 (Nrf2) is a key transcription factor involved in cellular defense mechanisms and in maintaining redox homeostasis, delaying cellular senescence and preventing age-related disorders [60,61]. When the activation of the Nrf2 pathway is insufficient to counteract the oxidative stress load, this may facilitate oxidative damage and the onset of senescence-associated responses [62].

Based on this evidence, we investigated whether the senescence-modulating effects of QUE could be associated with its ability to activate the Nrf2 pathway. Once activated, Nrf2 is released from its inhibitor Keap1 and translocates to the nucleus, where it binds to specific DNA sequences, known as Antioxidant Response Elements (AREs), and induces the transcription of several antioxidant mediators, such as NAD(P)H:quinone oxidoreductase 1 (NQO1) and heme oxygenase-1 (HO-1).

**Figure 8:**
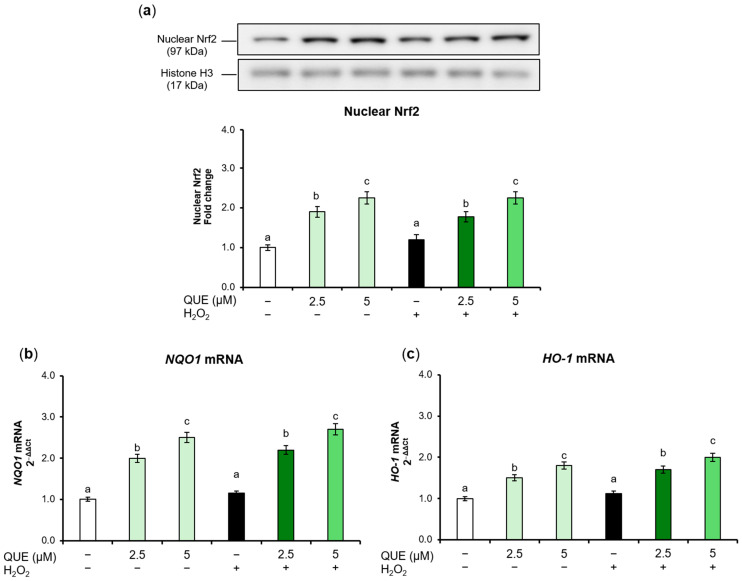
QUE activates the Nrf2/ARE pathway. SH-SY5Y cells were treated with H_2_O_2_ (25 μM) for 1 h, followed by incubation with QUE (2.5 and 5 μM) for 24 h. Cells treated with the vehicle alone (DMSO 0.01%) were used as controls. Nuclear Nrf2 (**a**) protein levels were determined in nuclear extracts, normalized to histone H3 levels and reported as fold change relative to control. *NQO1* (**b**) and *HO-1* (**c**) mRNA expression levels are expressed as 2^−ΔΔCt^, and normalized to controls; GAPDH was used as the housekeeping gene. Results are expressed as mean ± SD of three independent experiments, each performed in triplicate. Means sharing the same letter are not significantly different from each other (*p* > 0.05).

In our experimental model, QUE activated the Nrf2/ARE signaling pathway in cells exposed or not exposed to H_2_O_2_, as shown by the increased nuclear levels of Nrf2 (Figure 8a) and the upregulation of *NQO1* and *HO-1* gene expression (Figure 8b,c). Notably, the low-dose, short-term exposure to H_2_O_2_ used in our model (25 μM, 1 h) did not substantially induce the Nrf2/ARE pathway. Under these conditions, Nrf2 nuclear levels and the expression of both genes remained at basal levels, indicating that this moderate oxidative stress was not sufficient to elicit a detectable activation of the antioxidant response in SH-SY5Y cells. Overall, these findings suggest that Nrf2 activation is associated with the senoprotective effects of QUE in this model.

## 3. Discussion

Cellular senescence is a persistent and irreversible state of cell cycle arrest, which has gained increasing attention as a key biological mechanism linking aging, chronic diseases, and cancer [22]. As a result, targeting senescent cells represents a novel strategy to restore tissue homeostasis and improve age-related alterations [63]. Although cellular senescence has been extensively characterized in proliferative systems, the mechanisms and functional implications of SIPS in neuronal-like cells remain less defined.

Increasing evidence indicates that cellular senescence is a critical feature of brain aging as well as the onset and progression of NDs. The accumulation of senescent cells in the brain contributes to neurodegeneration through multiple mechanisms, including chronic neuroinflammation, disruption of neuronal microenvironment, impairment of mitochondrial function, and release of SASP factors [64,65]. Oxidative stress, particularly induced by H_2_O_2_ exposure, has been identified as a primary driver of SIPS in experimental models, especially in neuronal cells [11,66]. While replicative senescence results from the exhaustion of cellular proliferative capacity, SIPS induced by sub-lethal oxidative insults reproduces many senescent characteristics, including morphological alterations, early cell cycle arrest, increased SA-β-gal activity, and SASP activation [11].

QUE is one of the most abundant and pharmacologically active naturally occurring flavonoids in the human diet [67]. It exerts beneficial effects in models of neurodegenerative, cardiovascular, skeletal, metabolic, and ocular disorders, sharing molecular mechanisms related to aging, such as oxidative stress, chronic inflammation, mitochondrial dysfunction, impaired autophagy, and apoptosis [68,69,70,71]. At the molecular level, QUE modulates multiple aging-associated signaling pathways and has been widely investigated as a compound with reported senescence-related modulatory activities, including both senolytic and senomorphic properties, which may make it a promising candidate for targeting cellular senescence in neurodegenerative conditions [72,73,74]. Its senescence-modulating effects have been reported in different in vitro models of oxidative stress-induced senescence. In H_2_O_2_-induced senescent pre-adipocytes and adipocytes, QUE significantly reduced the number of SA-β-gal-positive cells, intracellular ROS levels, and the expression of pro-inflammatory SASP cytokines [28]. Similarly, in bone marrow mesenchymal stem cells exposed to H_2_O_2_, QUE stabilized heterochromatin structure, preserved heterochromatin integrity, and attenuated senescence-associated inflammation by inhibiting the retinoic acid–inducible gene I (RIG-I) signaling pathway [75]. However, the senescence-modulating effects of QUE in neuronal-like models of SIPS remain less explored.

The human neuroblastoma cell line SH-SY5Y is widely employed as an in vitro neuronal-like model, particularly in studies addressing neurodegenerative mechanisms, cellular stress responses, and the evaluation of potential therapeutic agents [76,77]. These cells are highly sensitive to senescence-inducing stimuli, including H_2_O_2_ exposure, which promotes the development of a senescence-like phenotype, reproducing key molecular and functional features observed in aging and neurodegenerative conditions. The present model reflects the induction of an early senescence-like phenotype following oxidative stress; however, it does not allow the assessment of long-term persistence or irreversibility of the senescent state. Therefore, the present study employed SH-SY5Y cells as an in vitro model to investigate oxidative stress-induced senescence-like responses and to evaluate the senescence-modulating potential of QUE in a neuronal-like context.

In our experiments, exposure to a sublethal H_2_O_2_ concentration (25 μM) significantly increased SA-β-gal activity [78,79]. Notably, QUE treatment markedly reduced SA-β-gal activity, suggesting mitigation of senescence-associated lysosomal dysfunction. This response is consistent with evidence showing that neuronal cells display a pronounced increase in SA-β-gal activity under oxidative conditions, a phenomenon linked to lysosomal expansion in neurons exposed to redox imbalance. Similarly, in SH-SY5Y cells, low to moderate H_2_O_2_ induces an increase in SA-β-gal-positive cells, confirming sensitivity to oxidative stress [80,81,82].

During senescence, lamin-B1 protein levels decline, leading to marked alterations in nuclear structure and chromatin organization, contributing to the establishment of cell cycle arrest and of SASP [37,83]. Additionally, lamin-B1 loss is implicated in several age-associated disorders, such as chronic obstructive pulmonary and NDs [35,84]. Our results showed that H_2_O_2_-induced senescence-like responses in SH-SY5Y cells promoted lamin-B1 downregulation, whereas QUE alleviated these senescence-associated nuclear lamina alterations. This observation aligns with evidence showing that oxidative and mitochondrial stress can destabilize the nuclear lamina in neuronal models [35,85].

Cell cycle arrest represents a protective response initiated by abnormal proliferative signals or stress factors that halt cell division [86]. Senescent cells typically exhibit a stable arrest in the G1 or G2 phases of the cell cycle, whereas quiescent cells remain reversibly in the G0 state and can resume proliferation upon appropriate stimuli [86]. This permanent proliferative arrest is regulated by tumour suppressor pathways, particularly the p53/p21^Cip1/Waf1^ axis, which inhibits CDKs and prevents cell-cycle progression [87]. In response to senescence-inducing factors, cells stabilize p53 and induce p21 transcription, blocking G1/S transition [87]. In contrast to the p16^INK4a^/pRB axis, which acts later to maintain irreversible arrest, the p53/p21 pathway is generally triggered early following acute stress [86]. In this model, QUE reduced activation of the p53/p21 axis in SH-SY5Y cells subjected to H_2_O_2_, demonstrating modulation of cell-cycle regulators. These findings are consistent with evidence that neuronal cells activate the p53/p21 pathway rapidly under oxidative stress, leading to a senescence-like cell-cycle arrest [88,89,90].

Although senescence and apoptosis are initiated by similar stress signals, including DNA damage, oxidative stress, and oncogenic activation, they represent two distinct processes. Apoptosis eliminates damaged cells through programmed cell death, whereas senescence maintains survival. Resistance to apoptosis is a hallmark of cellular senescence and contributes to the accumulation of dysfunctional cells during aging [91]. This shift is driven by an altered balance between pro-apoptotic (Bax) and anti-apoptotic (Bcl-2) proteins. SH-SY5Y cells exposed to H_2_O_2_ showed elevated levels of Bcl-2 and reduced Bax, increasing Bcl-2/Bax ratio and favouring cell survival. QUE modulated the balance between pro- and anti-apoptotic signaling, as indicated by a decreased Bcl-2/Bax ratio. Importantly, this modulation reflects changes in apoptosis-related signaling and does not provide direct evidence of functional apoptosis or selective elimination of senescent cells. These findings reflect the well-documented propensity of neuronal cells to develop apoptosis resistance under oxidative stress. Studies show that oxidative or mitochondrial stress promotes early anti-apoptotic shifts in neuronal cells, including increased Bcl-2 expression and reduced Bax levels [92,93].

The SASP represents a complex and dynamic secretome of pro-inflammatory cytokines, chemokines, growth factors, proteases, and extracellular matrix remodeling enzymes that influence the tissue microenvironment [94]. The SASP response triggers autocrine senescence, wound healing, and recruitment of immune cells; however, when senescent cells persist, chronic SASP causes tissue dysfunction, low-grade inflammation, and contributes to the pathogenesis of age-related diseases, such as neurodegeneration [95]. Moreover, senescent cells can transmit senescence to nearby normal cells, initiating paracrine senescence [96]. Recent proteomic studies have highlighted SASP components as promising plasma-based biomarkers of cellular senescence and age-related diseases [97]. In addition to senolytics that aim to eliminate senescent cells, SASP inhibitors or senomorphic agents represent alternative strategies to improve the senescence-associated phenotype [98]. The NF-κB pathway is the master regulator of SASP mediators, including pro-inflammatory interleukins and matrix metalloproteinases [49,94]. Among these, MCP-1 and PAI-1 have emerged as significant mediators of autocrine and paracrine senescence [54,57]. MCP-1 contributes to the maintenance and amplification of the senescent state, and p53 activation promotes MCP-1 secretion, establishing a positive feedback loop [54]. PAI-1 acts downstream of p53 and contributes to age-associated conditions [57,99]. Our results confirmed QUE inhibitory activity on SASP activation, as shown by reduced nuclear p65 levels and downregulation of *MCP-1* and *PAI-1* genes, selected as representative SASP-associated factors in this neuronal-like model. Similar modulation of senescence-associated inflammatory signaling has been reported in SH-SY5Y cells exposed to amyloid-β-induced oxidative stress, where pharmacological activation of the Nrf2/HO-1 axis reduced NF-κB p65 nuclear translocation and senescence markers [100]. The modest reduction of *MCP-1* and *PAI-1* observed following treatment is consistent with the complex, multilayered regulation of SASP factors. Indeed, SASP expression is driven not only by NF-κB but also by additional pathways, including p38 MAPK, ROS-dependent signaling, and TGF-β-associated circuits, reflecting its heterogeneous and context-dependent nature [94]. Moreover, neuronal cells are known to display a modest inflammatory SASP, and similar features have been described in neuronal-like models such as SH-SY5Y [101], which may explain the limited responsiveness of specific SASP genes to senescence-modulating interventions. In this context, the partial decrease of *MCP-1* and *PAI-1* integrates well with the broader senescence-modulating effects of QUE observed in our model. Importantly, the downregulation of PAI-1, a transcriptional effector of p53 and a mediator of p21-dependent cell cycle arrest, suggests that QUE may mitigate not only SASP-related inflammation but also aspects of senescence linked to the p53/p21–PAI-1 axis [56,57]. In addition to QUE, several compounds have been reported to modulate the SASP, including pharmacological agents such as metformin and the mTOR inhibitor rapamycin, as well as other senomorphic compounds. However, these agents often exert more selective effects on specific signaling pathways [102]. In contrast, QUE may act as a multitarget modulator, combining antioxidant and anti-inflammatory properties with the ability to influence multiple hallmarks of senescence. This multimodal profile may be particularly relevant in neuronal-like cells, where senescence is regulated by complex and interconnected pathways.

Given the modulation of key senescence markers, such as SA-β-gal activity, lamin-B1 expression, p53/p21 signaling, and SASP factors, we further explored the molecular basis of QUE’s protective role against H_2_O_2_-induced senescence, focusing on its antioxidant activity mediated by the Nrf2 pathway. As previously demonstrated, QUE can promote Nrf2 activation by inducing its nuclear translocation and binding to ARE elements [103], facilitated by either interference with the Keap1/Nrf2 complex [104] or Keap1 ubiquitination and degradation [105]. Through these mechanisms, Nrf2-dependent antioxidant and detoxifying genes, such as HO-1 and NQO1, are upregulated. Given these properties, Nrf2 represents a promising target for preventing age-related disorders, as selective Nrf2 activators have been reported to counteract cellular senescence [106,107,108], including in neuronal models of Alzheimer’s disease, where Nrf2 activation was shown to be required to suppress oxidative stress-induced senescence and NF-κB signaling [100]. In this experimental model, QUE activated the Nrf2 signaling pathway, as shown by nuclear localization of Nrf2 protein and upregulation of NQO1 and HO-1 gene expression, both in SH-SY5Y cells exposed or not to H_2_O_2._ This result supports an association between QUE treatment and the activation of ARE-dependent factors, thereby suggesting a contribution to the attenuation of oxidative stress-induced senescence in neuronal-like cells. Moreover, the cross-talk between Nrf2 and NF-κB may also be involved in QUE’s SASP-suppressing effects, possibly through the modulation of NF-κB-mediated inflammation [109,110,111]. Short-term exposure to low-dose H_2_O_2_ did not markedly activate the Nrf2–ARE pathway, and HO-1 and NQO1 expression remained at basal levels. This finding is consistent with the redox biology of Keap1–Nrf2, where significant oxidative modification of Keap1 is required to stabilize Nrf2 and trigger ARE-driven transcription, typically under stronger or more prolonged oxidative stress [112].

Overall, these observations, although derived from an in vitro model, are consistent with the broader interest in senescence-targeting strategies currently emerging in neurodegeneration research [31]. This study offers an integrated analysis of cell cycle regulators, oxidative stress-related pathways, and SASP-associated inflammatory mediators, highlighting their coordinated modulation in a neuronal-like context, where such combinatorial approaches remain relatively limited. The present model reflects the induction of an early senescence-like phenotype following oxidative stress; however, it does not allow the assessment of long-term persistence or irreversibility of the senescent state. Among the limitations of the present study, we must mention that this model of senescence induction is based on a single oxidative stress approach (low-dose, short-term H_2_O_2_), which reflects SIPS but may not fully represent the chronic oxidative stress linked to neurodegeneration. Although modulation of the p53/p21 axis is widely used as an indicator of cell cycle arrest, direct evaluation of cell cycle distribution or complementary functional assays was not performed. Therefore, cell cycle arrest was inferred from molecular and functional (SRB) data. Finally, while our findings suggest the involvement of Nrf2 and NF-κB pathways, mechanistic confirmation would require Nrf2 silencing or pharmacological modulation with specific inhibitors. Instead, our data indicate that Nrf2 is one of the redox-responsive pathways modulated by QUE in senescence-like SH-SY5Y cells. Future studies should extend these findings by validating the senescence-modulating effects of QUE in more physiologically relevant systems, such as primary neurons, and in longer-term studies that better mimic chronic oxidative stress and further define the contribution of redox-responsive pathways involved in its protective effects.

## 4. Materials and Methods

### 4.1. Reagents

Quercetin (≥98% purity) was purchased from Merck Life Science (Milan, Italy). 5-bromo-4-chloro-3-indolyl-β-D-galactopyranoside (X-gal) reagent was purchased from Merck Life Science (Milan, Italy). Hydrogen peroxide solution (H_2_O_2_) 3% (*v*/*v*) was obtained from Liofilchem S.r.l. (Teramo, Italy). All the other reagents, unless otherwise specified, were obtained from Merck Life Science (Milan, Italy).

### 4.2. Cell Cultures and Treatments

SH-SY5Y human neuroblastoma cells were acquired from American Type Culture Collection (ATCC, Manassas, VA, USA) and cultured in Roswell Park Memorial Institute (RPMI, Buffalo, NY, USA) 1640 medium, supplemented with 10% FBS, 2 mM L-glutamine and 100 U/mL penicillin/streptomycin. Cells were maintained at 37 °C in a humidified atmosphere with 95% air and 5% CO_2_. All experiments were performed using SH-SY5Y cells at passages 5–15, a range selected to avoid both low-passage variability and passage-associated senescence.

For the experiments, SH-SY5Y cells were seeded at a density of 0.8 × 10^5^ cell/cm^2^ in 12-well plates (Greiner Bio-One, Cassina de’ Pecchi, Italy) for gene expression analysis, and in 6-well plates (Greiner Bio-One, Italy) for protein studies. Subconfluent SH-SY5Y cells (~80% confluence, 2 days post-seeding) were exposed to 25 μM H_2_O_2_ for 1 h, followed by washing with DPBS containing calcium and magnesium. H_2_O_2_ was freshly prepared in RPMI medium immediately before use. This H_2_O_2_ exposure protocol was selected because it reliably induces an early senescence-like phenotype without causing evident cytotoxicity, as confirmed in preliminary experiments (Supplementary Materials, Figure S1), and is consistent with established stress-induced premature senescence models based on oxidative stress [113,114,115]. Subsequently, cells were treated for 24 h with QUE at two concentrations (2.5 and 5 μM). QUE was freshly dissolved in DMSO and used at non-cytotoxic concentrations as determined in preliminary experiments (Supplementary Materials, Figure S2); this QUE low-micromolar concentration range is commonly employed in SH-SY5Y and other neuronal-like models to elicit antioxidant and neuroprotective effects without impairing viability [116,117,118]. The final DMSO concentration in culture medium was maintained ≤0.01% (*v*/*v*). Cells treated with the vehicle alone (0.01% DMSO, *v*/*v*) were used as controls (CTR). Importantly, QUE treatment was applied after removal of the oxidant to ensure that its effects reflected modulation of the emerging senescent phenotype rather than direct chemical scavenging of H_2_O_2_, thus representing a post-treatment approach rather than pre- or co-treatment conditions and avoiding misinterpretation of its effects as simple antioxidant scavenging activity.

Following the treatments, SH-SY5Y cells were detached with Trypsin-EDTA and stored at −20 °C for subsequent analysis.

### 4.3. Senescence-Associated-β-Galactosidase Staining Assay

The senescence-associated-β-galactosidase (SA-β-gal) staining assay was performed to detect senescent cells based on β-galactosidase activity at pH 6.0, as previously described by Itahana et al. [119].

To improve morphological resolution during imaging, all samples designated for SA-β-gal analysis were seeded at a lower density (~50% confluence at the time of H_2_O_2_ exposure). This adjustment ensured homogeneity across experimental groups and avoided density-dependent artifacts, while still maintaining identical H_2_O_2_ exposure conditions for all samples. At the H_2_O_2_ sub-cytotoxic dose used (25 µM for 1 h), SH-SY5Y cells do not exhibit density-dependent differences in early oxidative injury, as confirmed in preliminary viability assessments (Supplementary Materials, Figure S1).

At the end of appropriate treatments, SH-SY5Y cells were first washed twice with DPBS with calcium and magnesium and then fixed with neutral buffered 4% (*v*/*v*) formaldehyde for 3 min at room temperature. Subsequently, 1.5 mL of SA-β-gal staining solution (1 mg/mL 5-bromo-4-chloro-3-indolyl-β-D-galactopyranoside (X-gal), 1× citric acid/sodium phosphate buffer (pH 6.0), 5 mM potassium ferricyanide, 5 mM potassium ferrocyanide, 150 mM NaCl, and 2 mM MgCl_2_) was added to each well, and the cells were then incubated at 37 °C in a non-CO_2_ incubator. After 16 h of incubation, blue staining was fully developed, the staining solution was removed, and cells were washed twice with DPBS. A drop of mounting medium or DPBS was added, and finally, stained cells were observed using an inverted microscope and photographed at 20× magnification. For quantification, SA-β-gal-positive cells were counted in five randomly selected fields per condition from three independent experiments. Images were converted to 8-bit in Fiji/ImageJ (version 1.54p), and contrast was linearly adjusted (0.3–0.5% saturated pixels). A threshold-based segmentation was applied to identify β-gal-positive cells, followed by Analyze Particles to obtain the number of positive events. The total number of cells in each field was determined from the corresponding bright-field image, and results are expressed as the percentage of SA-β-gal-positive cells relative to the total cell count. All thresholding and image-processing steps were applied uniformly across conditions within each experiment.

### 4.4. Cells Lysate Extraction

Whole cell lysates were obtained using a lysis buffer (10 mM Tris-HCl, pH 7.4, 150 mM NaCl, 1% Triton X-100, 5 mM EDTANa_2_). To acquire nuclear extracts, cells were first incubated with a hypotonic buffer (10 mM HEPES, pH 7.9, 10 mM KCl, 1.5 mM MgCl_2_, and 5% glycerol) to separate cytoplasmatic proteins, and subsequently with a hypertonic buffer (20 mM HEPES, pH 7.9, 1 mM MgCl_2_, 400 mM NaCl, 1 mM EGTA, 0.1 mM EDTA, and 10% glycerol) for nuclear ones. All lysis buffers were supplemented with protease inhibitors (1 μg/mL leupeptin, 1 mM benzamidine, and 2 μg/mL aprotinin) and 1 mM dithiothreitol (DTT). At the end, protein lysates were stored at −20 °C until analysis. Protein concentrations were measured through the Bradford reagent [120], using bovine serum albumin as a standard.

### 4.5. Western Blot Analysis

For Western blot analysis, whole cell lysates (30 μg) or nuclear ones (10 μg) per sample were denatured in 4× SDS-PAGE sample buffer and separated by electrophoresis as previously described by Molonia et al. [121].

For immunoblotting, membranes were treated overnight at 4 °C with specific primary antibodies: rabbit anti-phosphor-p53 (Ser15) polyclonal antibody (Cell Signaling Technology, Danvers, MA, USA) (1:1000); mouse anti-p21^Waf1/Cip1^ monoclonal antibody (Santa Cruz Biotechnology, Dallas, TX, USA) (1:500); rabbit anti-Bax monoclonal antibody (Cell Signaling Technology) (1:1000); rabbit anti-Bcl-2 polyclonal antibody (Sigma-Aldrich, St. Louis, MO, USA) (1:1000); rabbit anti-NF-kB p65 monoclonal antibody (Cell Signaling Technology) (1:1000); rabbit anti-Nrf2 polyclonal antibody (1:1000) (Cell Signaling Technology), rabbit anti-Lamin-B1 monoclonal antibody (Cell Signaling Technology) (1:1000); rabbit anti-β-actin monoclonal antibody (Cell Signaling Technology) (1:6000); rabbit anti-histone H3 polyclonal antibody (Cell Signaling Technology) (1:1000). Subsequently, membranes were washed three times with TBST and incubated for 2 h at 4 °C with peroxidase-conjugated secondary antibody: anti-rabbit Ig (Invitrogen, Milan, Italy) (1:6000) or anti-mouse Ig (Cell Signaling Technology) (1:6000).

Protein bands were visualized using the Clarity Max ECL Substrate (Bio-Rad, Hercules, CA, USA) and captured with a ChemiDoc Imaging System (Bio-Rad, Hercules, CA, USA). Equal protein loading was verified with Ponceau S staining. β-actin and histone H3 were used as reference proteins for whole or nuclear fractions, respectively. Densitometric quantification was carried out using the Image Lab software (Version 6.1.0, build 7, Bio-Rad, Hercules, CA, USA).

### 4.6. Real-Time PCR

Total RNA was isolated using the E.Z.N.A. Total RNA Kit (Omega Bio-tek, Inc, Norcross, GA, USA), allowing the manufacturer’s instructions, and quantified through Quant-iT™RNA assay kit, using a QUBIT fluorometer (Invitrogen). Complementary DNA (cDNA) was transcribed with an M-MLV reverse transcriptase. Quantitative real-time PCR (polymerase chain reaction) analysis was performed using SYBR green chemistry (SYBR green JumpStart^TM^ Taq Ready Mix, Sigma-Aldrich, St. Louis, MO, USA) on the 7300 Real-Time PCR System (Applied Biosystems, Monza, Italy). The following primers were used:

*p53* (FW: 5′-CCTCAGCATCTTATCCGAGTGG-3′, RV: 5′-TGGATGGTGGTACAGTCAGAGC-3′), *p21* (FW: 5′-AGGTGGACCTGGAGACTCTCAG, RV: 5′-TCCTCTTGGAGAAGATCAGCCG-3′) [122]; *MCP-1* (FW 5′-CAGCCAGATGCAATCAATGCC-3′, RV 5′-TGGAATCCTGAACCCACTTCT-3′); *PAI-1* (FW 5′-ACCGCAACGTGGTTTTCTCA-3, RV 5′-TTGAATCCCATAGCTGCTTGAAT-3′) [123]; *NQO1* (FW 5′-AAGAGCACTGATCGTACTGG-3′, RV 5′-CTTCAGTTTACCTGTGATGTCC-3′); *HO-1* (FW 5′-CAACATCCAGCTCTTTGAGG-3′, RV 5′-AGAAAGCTGAGTGTAAGGAC-3′) [124]; *GAPDH* (FW 5′-GGCTCTCCAGAACATCATCCCTGC-3′, RV 5′-GGGTGTCGCTGTTGAAGTCAGAGG-3′) [125] served as the housekeeping gene. The relative mRNA expression levels were determined with the 2^−ΔΔCt^ method [126], normalized to GAPDH, and expressed as fold change compared with the CTR.

### 4.7. Statistical Analysis

The experiments were conducted in at least three independent biological experiments (n = 3). Data were analysed as follows: dose–response experiments (H_2_O_2_ or QUE alone) were evaluated by one-way ANOVA, followed by Tukey’s HSD. Experiments involving combined treatments were analysed by two-way ANOVA with H_2_O_2_ exposure and quercetin concentration as independent factors, followed by Tukey’s HSD. Statistical analyses were performed using the ezANOVA software (version 0.985, University of South Carolina, Columbia, SC, USA). The software is available at https://people.cas.sc.edu/rorden/ezanova/index.html (accessed on 10 November 2025). Differences between groups and treatments were considered statistically significant at *p* < 0.05.

## 5. Conclusions

The present study shows that H_2_O_2_-induced oxidative stress effectively promotes an early SIPS phenotype in SH-SY5Y neuronal-like cells, as evidenced by increased SA-β-gal activity, lamin-B1 alterations, modulation of p53/p21 signaling, changes in Bcl-2/Bax balance, and activation of SASP-related inflammatory response. Moreover, QUE exhibited a protective effect against H_2_O_2_-induced senescence-like response by attenuating key markers of cellular senescence. Particularly, it enhanced the Nrf2/ARE antioxidant response, which was associated with cytoprotective defense mechanisms. These observations, although derived from an in vitro model, are consistent with the broader interest in senescence-targeting strategies currently emerging in neurodegeneration research.

Overall, these findings highlight the activity of QUE as a senescence-modulating agent capable of modulating multiple factors involved in neuronal senescence and may contribute to limiting the progression of age-related diseases.

## Data Availability

The data presented in this study are contained within the article or Supplementary Materials. The raw data supporting the conclusions of this study are available from the corresponding author upon reasonable request.

## Supplementary Materials

The following supporting information can be downloaded at https://www.mdpi.com/article/10.3390/ijms27135759/s1. References [125,127] are cited in the Supplementary Materials.

## Abbreviations

The following abbreviations are used in this manuscript:

SASP	Senescence-associated secretory phenotype
QUE	Quercetin
SIPS	Stress-induced premature senescence
SA-β-gal	Increased senescence-associated β-galactosidase
NDs	Neurodegenerative diseases
