# Supplementary material for: Quercetin Mitigates Oxidative Stress-Induced Premature Senescence in SH-SY5Y Neuronal-like Cells
Source: Int J Mol Sci. 2026 Jun 26;27(13):5759. doi: 10.3390/ijms27135759 (PMC13360623; doi:10.3390/ijms27135759)
Supplement: Supplementary file 1 [file ijms-27-05759-s001.zip › ijms-4370740-supplementary.pdf]

Supplementary material

Quercetin Mitigates Oxidative Stress-Induced Premature Senescence in SH-SY5Y Neuronal-like Cells

Federica Lina Salamone, Maria Sofia Molonia, Santi Trischitta, Antonella Saija, Francesco Cimino and Antonio Speciale

In preliminary experiments, we evaluated H_2O_2 and quercetin (QUE) cytotoxicity on SH-SY5Y cells through the Sulforhodamine B (SRB) assay (Vichai and Kirtikara, 2006). Following the treatments, cells were fixed using 10% (w/v) trichloroacetic acid for 1 hour at 4°C, rinsed twice with distilled water and subsequently stained with sulforhodamine B (SRB; 0.4% w/v in 1% acetic acid) for 30 minutes at room temperature in the dark. SRB residues were removed by washing the cells with 1% acetic acid. The retained dye was solubilized in a 10 mM Tris-base solution, and absorbance was measured at 565 nm using a microplate reader (GloMax® Discover System-TM397). Cell viability, as determined by the SRB assay, was expressed as a percentage (%) relative to CTR cells.

SH-SY5Y cells were exposed to H_2O_2 (10, 25, 50, and 100 μM) for 1 hour, followed by 24 hours in fresh medium. Results indicated that H_2O_2 induced significant cytotoxicity starting at 50 μM concentration (Fig. S1). Thus, for further analysis, 25 μM H_2O_2 was selected as a sub-cytotoxic concentration, minimizing acute cytotoxic effects while enabling investigation of early senescence-associated cellular responses characterized by mild growth inhibition in our experimental model.

Under these conditions, exposure to H_2O_2 (25 μM) resulted in a modest increase in intracellular ROS levels (114 ± 3% vs CTR), consistent with the induction of a mild oxidative imbalance that may contribute to downstream senescence-related responses. Intracellular ROS levels were evaluated using the DCFH-DA assay, as previously described (Salamone et al. 2025). SH-SY5Y cells were exposed to H_2O_2 (25 μM) for 1 h, and ROS values were normalized to protein content and expressed as percentage (%) relative to control (CTR).

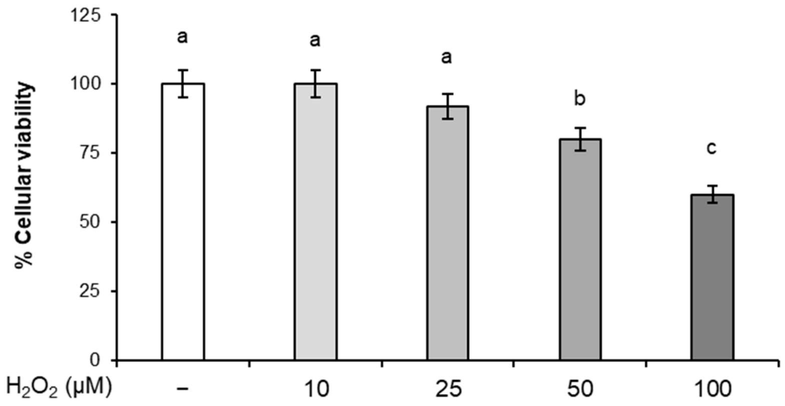

Figure S1. Effects of H_2O_2 on cell viability evaluation. SH-SY5Y cells were exposed to H_2O_2 (10, 25, 50 and 100 μM) for 1 hour, followed by 24 hours in fresh medium. Cells treated with the vehicle alone (DMSO 0.01%) were used as controls. Results are reported as the percentage of cell viability against controls (%) and expressed as mean ± SD. Means with the same letter are not significantly different from each other ($p > 0.05$).

For QUE cytotoxicity evaluation through the SRB assay, cells were treated with QUE at different concentrations (from 1 to 50 μM) for 24 hours, and cell viability was assessed by SRB assay as described above. As shown in Fig. S2, QUE treatment at 2.5 and 5 μM did not significantly affect cell viability in SH-SY5Y cells. In contrast, higher concentrations (25 and 50 μM) significantly reduced cell viability, while 10 μM induced a mild, non-significant viability decrease. Based on these results, 2.5 and 5 μM were selected for subsequent experiments.

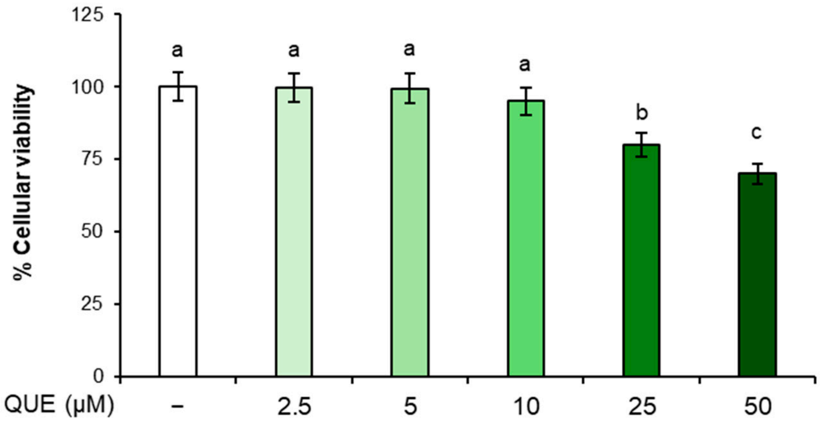

Figure S2. Effects of QUE on viability. SH-SY5Y cells were treated with QUE (2.5, 5, 10, 25, and 50 μM) for 24 hours. Cells treated with the vehicle alone (DMSO 0.01%) were used as controls. Results are reported as the percentage of cell viability against controls (%) and expressed as mean ± SD. Means with the same letter are not significantly different from each other ($p > 0.05$).

SRB assay was also used to assess the effects of QUE treatment following H_2O_2 exposure. As shown in Fig. S3, treatment with QUE (2.5 and 5 µM) following 25 µM H_2O_2 exposure did not induce a decrease in cell number. These findings support the interpretation that the observed modulation of molecular markers is not associated with non-specific cytotoxicity.

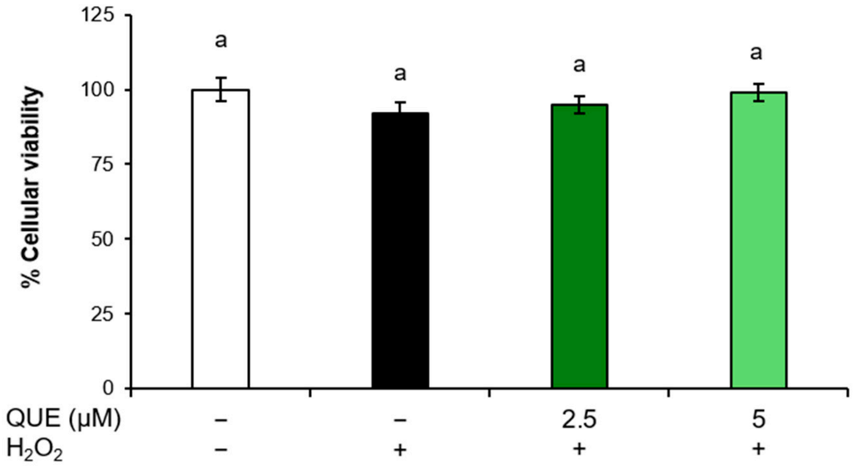

Figure S3. Effects of H_2O_2 and QUE treatment assessed by SRB assay. SH-SY5Y cells were exposed to H_2O_2 (25 µM) for 1 hour, followed by washing and treatment with QUE (2.5 and 5 µM) for 24 hours. Cells treated with the vehicle alone (DMSO 0.01%) were used as controls. Results are reported as the percentage of cell proliferation relative to controls (%) and expressed as mean ± SD. Means with the same letter are not significantly different from each other ($p > 0.05$).

REFERENCES

- Salamone, F. L.; Molonia, M. S.; Trischitta, S.; Saija, A.; Cimino, F.; Speciale, A., Continuous exposure to low concentrations of antimony(III) induces inflammation, apoptosis, oxidative and endoplasmic reticulum stress in Caco-2 intestinal epithelial cells. *Environ Res* 2025, 281, 122001.
- Vichai, V.; Kirtikara, K., Sulforhodamine B colorimetric assay for cytotoxicity screening. *Nat Protoc* 2006, 1, (3), 1112-6.
